# Communication from Dr. D. Genese

**Published:** 1884-08

**Authors:** D. Genese


368 MADISON AVE., BALTIMORE, *July 25th,* 1884.

MR. EDITOR:—The Edition of the *Dental Advertiser* for July doubts my statenent respecting (dovetaily and undercuts in Vulcanite rubber being tought in schools or Dental Works on Mechanism.

As I am a stranger in this part of the world I can only refer him to Charles James Fox, the present Editor and Walter Blundell, the first Editor of *British Journal of Dental Science*, England, for both of whom I did work as described in 1862, and was noted in Journals about that time, and as we are upon the subject of instruction in Mechanical Dentistry and matters bearing thereon, may I ask how is it that a patent has been granted within the last year for (bleaching rubber plates by exposure to solar light in alcohol) to an American dentist when Ash, of London have published the process in their catalogue as long as dental rubbers have been in use, and which should be taught in every dental laboratory.

The dentist making oath that he is the original inventor of the process must have taken advantage of the *omission* on the part of (Editors authors and professors on this side of the Atlantic to instruct the students in some of the older methods of Dental Mechanism but which are still considered important enough to take a patent upon.

Some dovetail work was shown by me at the Deer Park Convention, and at Philadelphia on a former visit in 1877, and among other things I brought over for inspection was *articulating* paper which I see announced in this months *Cosmos* as patented here, but which is the old form of manifold writing employed by drapers in England, and prepaired expressly for dental use by Field, of London, under a patent of Mr. Barkley, of same place. Sold at 50c. a dozen, but pirated on this side and retailed at $1.00

If the Society that Dr. Catching, of Atlanta, is trying to form once gets started it will be a great gain to the profession.

The idea is that it should be a truely National one, with a Museum at Washington, containing the records of every known appliance in dentistry with model or drawing of same, preparations of materials used in past and present library and books on dentistry to be collected from all parts of this country and abroad, and a periodical record sent in from every State Dental Society, which together would be of vast importance to the examiners at the Patent Office, and protect the profession from——that wholesale filching of property which so often occurs, caused no doubt by the vast distances that may intervene between the original inventor who may only be a visitor here, or show his design at some convention, and the unscrupulous person that may chance to see or hear of it from the reports in our journals and which do not find their way to the Patent Office at Washington.

D. GENESE.

